# Social distance of bystanders affects people’s embarrassment via changing fear of negative evaluation and feelings of attachment security

**DOI:** 10.1186/s40359-023-01201-7

**Published:** 2023-05-18

**Authors:** Hongjuan Tang, Lin Li, Li Zheng, Xiuyan Guo, Haoyue Qian

**Affiliations:** 1 School of Education, XiZang MinZu University, Xianyang, Shaanxi 712082 China; 2 School of Psychology and Cognitive Science, East China Normal University, Shanghai, 200062 China; 3 Fudan Institute on Ageing, Fudan University, Shanghai, 200433 China; 4 MOE Laboratory for National Development and Intelligent Governance, Fudan University, Shanghai, 200433 China; 5 Institute of Neuroscience, Key Laboratory of Primate Neurobiology, CAS Center for Excellence in Brain Science and Intelligence Technology, Chinese Academy of Sciences, Shanghai, 200031 China; 6 CAS Center for Excellence in Brain Science and Intelligence Technology, Chinese Academy of Sciences, Shanghai, 200031 China

**Keywords:** Embarrassment, Bystander, Social distance, State attachment security, Self-conscious emotion

## Abstract

**Background:**

Embarrassment is a self-conscious emotion with important social functions, but it is not well understood. The perception of bystanders is considered a precondition for embarrassment, which makes it unique from other self-conscious emotions. Studies have shown that socially close bystanders can reduce individuals’ embarrassment. However, whether and how the embarrassment of individuals varies with the changes in social distance between them and their bystanders remained unclear, which indicates the key characteristics of embarrassment.

**Methods:**

The current research consists of two studies. Study 1 tested whether participants’ embarrassment systematically varied with social distance by setting up three levels of social distance: close friends (i.e., short), casual friends (i.e., medium), and strangers (i.e., long), based on 159 participants. With two full mediation models, study 2 investigated whether and how the fear of negative evaluation and state attachment security mediated the influence of social distance on embarrassment based on 155 participants.

**Conclusions:**

The current findings revealed that the social distance between bystanders and protagonists systematically influenced the embarrassment of protagonists and this effect occurred via two parallel pathways, i.e., by increasing the fear of negative evaluation and by reducing state attachment security. The findings not only showed the unique role of bystander characteristics on embarrassment, but also two cognitive processes behind this unique self-conscious emotion: fearing negative evaluation and seeking attachment for security.

**Supplementary Information:**

The online version contains supplementary material available at 10.1186/s40359-023-01201-7.

## Background

Embarrassment refers to the flustering occurring due to a perceived fumbled performance [1], which is a unique self-conscious emotion [2, 3]. However, in contrast to other negative self-conscious emotions such as shame and guilt, embarrassment has been investigated by a small number of studies and was previously even considered to have a large overlap with shame. There is growing evidence that the perception of real or imagined bystanders makes embarrassment special and distinct from other negative self-conscious emotions, such as shame. For example, an increase in the number of bystanders increased embarrassment [4] but did not affect shame [5]. What is more, some researchers argue that the presence of imagined or real others is a prerequisite for embarrassment [4, 6], but only a facilitator for shame. That is, there is shame alone, but not embarrassment alone [7, 8]. Therefore, studying how the characteristics of bystanders, such as their social distance from the protagonist, affect embarrassment can help us to understand embarrassment as a unique emotion.

Research has showed that when bystanders around them were family members or friends (compared to strangers or new acquaintances), both children and adults experienced less embarrassment [9, 10]. These findings suggested that individuals’ embarrassment was influenced by the social distance between them and the bystanders present, i.e., the psychological distance perceived by individuals between themselves and others [11–13]. Nevertheless, previous studies always addressed this issue by comparing two conditions, and few studies examined how embarrassment changes with social distance by setting several sequential variation levels. Thus, the influence of the social distance on embarrassment was not well verified.

More importantly, how social distance affects embarrassment has not been investigated, whose result is important for the understanding of the cognitive process underlying embarrassment. According to the social evaluation theory [14], fear of negative evaluation is one of cognitive process underlying embarrassment and should be considered. Specifically, the occurrence of unexpected misconduct may make people worry that bystanders will negatively evaluate their public image and further feel embarrassment. However, when the bystander is socially close to them (e.g., a family member or friend), the embarrassed individuals would believe that the bystander already has a stable impression of them and would not judge them negatively simply due to an embarrassing incident, and reduce their worries and then feeling of embarrassment. Thus, we expected that short social distance might reduce the cognitive process associated with evaluation fear from bystanders. That is, fear of negative evaluations from others) may be a mediator of the effect of social distance on embarrassment.

On the other hand, feeling of attachment security may be closely related to embarrassment, although it has often been overlooked before. Research has shown that a close relationship between people’s feelings of embarrassment and their real-time insecure or anxious state [15, 16]. When facing an embarrassing event, individuals with higher anxiety were more likely to experience embarrassment [17]. That is, the intensity of individuals’ embarrassment may depend on their feelings of being insecure or unsettled. Attachment theory suggests that the presence of a close person temporarily activates the attachment system of a flustered individual and allows them to feel secure from their attachment object [18]. Correspondingly, if the bystander is socially close, the embarrassed individual’s momentary sense of security would be enhanced due to the attachment finding, referred to as state attachment security [19], and further this enhanced attachment security would reduce their feeling of embarrassment. Note that, the attachment system of flustered individuals may be activated immediately after the embarrassing event, or after feeling fear of negative evaluation due to the embarrassing event. Given the above, we could not give a specific hypothesis on the interplay of state attachment security and fear of negative evaluation. Collectively, we expected that state attachment security may be a second mediator in the effect of the social distance on embarrassment, while its interplay with fear of negative evaluation remained to be explored.

Briefly, we planned to set up two studies to investigate whether and how social distance from bystanders affects embarrassment. Study 1 tested whether participants’ embarrassment systematically varied with social distance by setting up three levels of social distance. Using two full mediation models, study 2 further examined whether and how the fear of negative evaluation and state attachment security mediate the effect of social distance. The answer could help us to further understand not only the effect of bystander on embarrassment, but also relevant cognitive process underlying the feeling of embarrassment.

### Study 1: varying embarrassment as the changes in social distance

#### Methods

##### Participants

Using the G-Power software, we found that a sample size of 159, i.e., 53 subjects in each condition, could ensure that a medium-sized experimental effect could be observed (Power = 0.80). Accordingly, 159 college students (mean age = 21.33 years, *SD* = 0.85; 138 females) were recruited to participate in the study 1. Participants were recruited through the online subject collection platform of the University. All participants were native Chinese speakers with normal or corrected-to-normal vision.

#### Material

##### Embarrassment scenarios

The first study used textual materials to induce embarrassment. Specifically, it selected 10 embarrassment scenarios, e.g., falling in public, greeting and recognizing the wrong person, and forgetting lines when hosting a program (see Supplemental materials S1) according to the previous literature [20, 21]. All embarrassing scenarios were set according to everyday life, where there were several bystanders. During the test, participants viewed each embarrassing scenario as a vignette for 10 s and were required to imagine being the protagonist with a close friend (or casual friend, or stranger) and some other stranger bystanders. Prior to the test, each participant was asked to recall a gender-matched close friend and a gender-matched casual friend and to write down their names, and these names were present in the embarrassing scenarios of the test. The “imagery” design was applied to induce a more vivid and realistic experience of embarrassment, and the “gender-matched” design was to control the potential confounding effect of gender on embarrassment [22].

##### Social distance measurement

In the study 1, we applied 3 kinds of bystanders with different social distances from the participants, i.e., close friends, casual friends, and strangers. The social distances between the embarrassed protagonists (i.e., participants themselves) and different kinds of bystanders were examined using the Inclusion of Other in the Self (IOS) scale [23]. This scale includes seven paired circles; one of the paired circles represents the participant, while the other one represents “other” (i.e., the bystander). The overlapping degrees of the paired circles linearly increase from left to right, and a larger overlap indicates increased closeness between two people.

##### Embarrassment measurement

For each embarrassment scenario, participants needed to quickly score their embarrassment with a scale ranging from 1 (not at all) to 7 (very embarrassed). The mean score of each participant’s embarrassment ratings in 10 scenarios was used to indicate the embarrassment intensity (Cronbach’s α = 0.88).

#### Procedures

This study has been approved by the University Committee on Human Research Protection of the East China Normal University (HR 083-2018). Before the experiment, written informed consent was obtained from each of participants. First, participants were asked to write down the names of both a gender-matched close friend and a gender-matched casual friend and then complete social distance measurement. Next, participants were randomly divided into the following three conditions: short social distance (i.e., close friend), medium social distance (i.e., casual friend), and long social distance (i.e., stranger). The participants of the short social distance (or medium social distance) condition read the following instruction on screen: “In the test, you will read 10 short stories. You are the protagonist of these stories, and one of the bystanders is a close friend (or casual friend), and the others were strangers.“ The participants of the long social distance condition read the instruction: “In the test, you will read 10 short stories. You are the protagonist of these stories, and the bystanders were strangers.“ After each story, participants were asked to rate their level of embarrassment. The task began when participants pressed the “space” button. The mean score of each participant’s embarrassment ratings in 10 scenes was used to indicate the embarrassment intensity (Cronbach’s α = 0.88). After the test, participants were paid 60 Chinese Yuan per hour.

## Results

### Measurement of social distance

In study 1, there were 53 participants in each condition (short, medium, or long social distance), and they were of similar ages (mean:*C*_*1*_ = 21.36, *C*_*2*_ = 21.26, and *C*_*3*_ = 21.35) *and gender ratios (*female: *C*_*1*_ = 0.85, *C*_*2*_ = 0.91, and *C*_*3*_ = 0.85). First, we examined whether the reported social distance differed across the three conditions. We found that the participants from the long social distance condition all reported the largest social distance (*M* = 7, *SD* = 0). Given the SD is zero, a standard ANOVA test cannot be applied to the comparison of the three conditions here. We used a Bonferroni-corrected *t*-test to test the differences between each of the two conditions. The results revealed the social distance between the key bystander present in the materials and the participants was different across the three conditions (close friend vs. stranger: *t* (52) = 36.88, *p* < .001, Cohen’s *d* = 7.15; causal friend vs. stranger, *t* (52) = 12.10, *p* < .001, Cohen’s *d* = 2.36; close friend vs. causal friend, *t* (104) = 19.41, *p* < .001, Cohen’s *d* = 3.76). These results indicated the manipulation of social distance was effective (see Table 1).

**Table 1:** Participants’ ratings of three conditions (short, medium, and long social distance)

	Condition	SD	EMB	FNE	SAS
Study 1	Short (Close friend)	2.25 ± 0.94	2.58 ± 0.97		
	Medium (Causal friend)	5.60 ± 0.84	3.97 ± 1.21		
	Long (Stranger)	7.00 ± 0	4.46 ± 0.84		
Study 2	Short (Close friend)	2.11 ± 1.14	2.73 ± 1.03	2.51 ± 1.08	4.94 ± 1.55
	Medium (Causal friend)	5.07 ± 1.14	3.87 ± 0.81	3.63 ± 0.94	3.47 ± 1.09
	Long (Stranger)	7.00 ± 0	4.36 ± 1.05	3.75 ± 1.07	2.51 ± 0.96

Note: SD = Social distance (ranging from 1 to 7); EMB = Embarrassment (ranging from 1 to 7); FNE = Fear of negative evaluation (ranging from 1 to 7); SAS = State attachment security (ranging from 1 to 7)

#### Effect of social distance on embarrassment

**Fig. 1:**
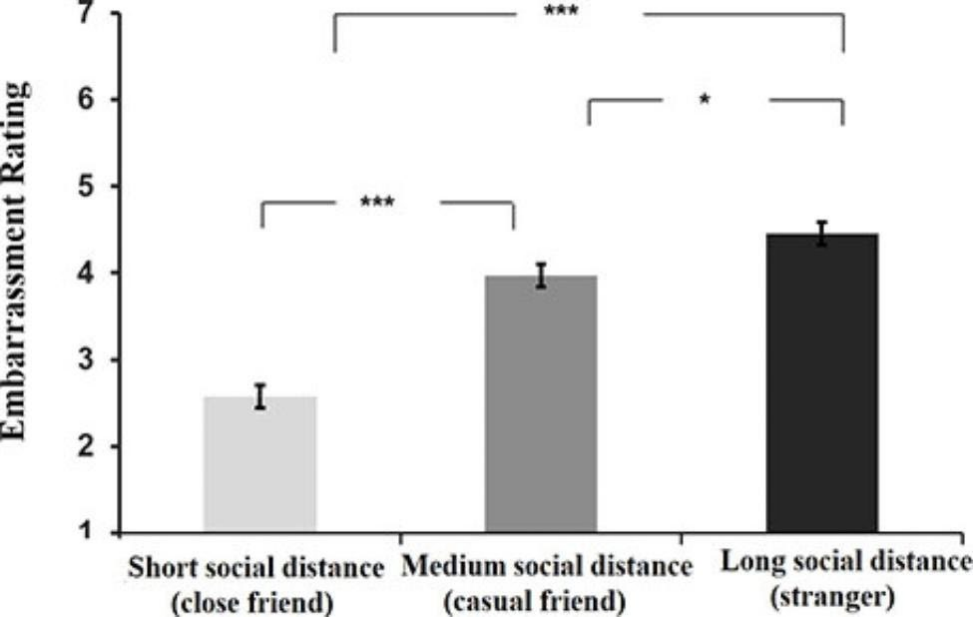
**Embarrassment ratings as a function of social distance in the study 1.** Participants reported the highest level of embarrassment when facing strangers (i.e., long social distance condition), the second-highest level when facing casual friends (i.e., medium social distance condition), and the lowest level when facing close friends (i.e., short social distance condition). Error bars indicate the standard error of the mean. ***p < .001. *p < .05

To examine whether the embarrassment of participants was modulated by the social distance between them and the bystander, we conducted a one-way analysis of variance (ANOVA) on the embarrassment scores with social distance as a between-subjects factor (Long, Medium, and Short social distance conditions). The results revealed a significant main effect of social distance on participants’ embarrassment, *F* (2, 156) = 48.69, *p* < .001, *η*_*p*_^2^ = 0.38. Further analysis revealed significant differences between each pairing condition, *ps* < 0.05 (Bonferroni corrected). Specifically, consistent with our hypothesis, participants reported the highest level of embarrassment when facing a stranger (*M* = 4.46, *SD* = 0.84), the second-highest level when facing a casual friend (*M* = 3.97, *SD* = 1.21), and the lowest level when facing a close friend (*M* = 2.58, *SD* = 0.97) (see Fig. 1). These findings suggest that the embarrassment of participants systematically varied with different degrees of social distance between them and bystanders.

#### Study 2: mediating factors underlying the social distance influencing embarrassment

In the study 1, we confirmed the levels of embarrassment varied with the changes in the social distance between embarrassed individuals and bystanders. In the study 2, we used mediation analyses to further investigate the potential mediators in the effect of the social distance on embarrassment. Specifically, we focused on whether fear of negative evaluations and state attachment security were two mediators and, if so, how the two mediators worked.

#### Participants

According to the effect size of the study 1 (*η*_*p*_^2^ = 0.38), 30 participants were required to observe the significant effect of social distance (Power = 0.80). To ensure that the mediators of social distance affecting embarrassment could be measured, study 2 still used a relatively large sample size similar to that of the study (1) Thus, 155 college students (mean age = 19.25 years, *SD* = 0.92; 66 females) were recruited to participate in the study (2) Participants were recruited through the online subject collection platform of the university. All participants were native Chinese speakers with normal or corrected-to-normal vision. Data from four participants in this study were excluded from the analyses due to an error in the operation of the computer program.

#### Materials

##### Measurement of real-time fear of negative evaluation

In this study, we planned to measure participants’ feelings of negative evaluation concerns with the classical fear of negative evaluation (FNE) scale [24, 25]. Notably, we needed to measure how participants felt in each scenario and the full version of the scale is too long to use. Thus, we decided to choose representative questions from the classical FNE scale to measure participants’ real-time feelings of fear of negative evaluation. To address this issue, we recruited 22 students prior to the experiment 2 to select the representative questions from the FNE scale. According to their ratings, only two questions (“I worry about leaving a bad impression on others”, and “I worry about what others think about me, even though I know that others’ thoughts are of little importance”) were consistently considered as the representative questions by over 90% (n = 20) of students. The two questions used here are from the Chinese version of the scale, called General Survey of Personality and Social Psychometric Measures [26]. Finally, these two questions were selected as the testing questions, and the phrase “at this moment” was added to the beginning of each question to indicate to participants that they should report their real-time feelings during the study 2. The mean score of participants’ responses to the two questions was used to indicate the levels of their real-time fear of negative evaluation; the scores ranged from 1 (not at all) to 7 (very much) (Cronbach’s α = 0.96).

#### Measurement of real-time feelings of state attachment security

The approach of measuring real-time feelings of state attachment security is inspired by Mikulincer and Shaver [27], which has been validated and applied by the previous study [28]. Specifically, four words expressing a sense of attachment security were used to the construct questions, “At this moment, when I imagine a close friend (or casual friend, or stranger) around me, I feel warm/ safe/ supported/ being cared for as the following extent: _____”. Participants were asked to answer these four questions on a 7-point scale, with responses ranging from 1 (not at all) to 7 (very much). The mean score of the four questions was used to indicate the level of the state attachment security of participants (Cronbach’s α = 0.97).

#### Procedures

This study has been approved by the University Committee on Human Research Protection of the East China Normal University (HR 083-2018). Before the experiment, written informed consent was obtained from each of the participants. The procedures of the study 2 were the same as those of the study 1, except that participants needed to rate their fear of negative evaluation and state attachment security before rating their embarrassment in each embarrassing scenario. The order of rating tasks for each scenario was as follows: state attachment security score, fear of negative evaluation score, and embarrassment score. Notably, the embarrassing scenarios were presented on a screen while participants completed all rating questions on paper. This approach was adopted to prevent the diminishing of the effect of the embarrassing situation on participants over time. After the study, participants were paid 60 Chinese Yuan per hour.

## Results

### Measurement of social distance

In study 2, there were 151 participants in the three conditions (short social distance: 54, medium social distance: 46, long social distance: 51). They were similar in ages (mean: *C*_*1*_ = 18.94, *C*_*2*_ = 19.63, *C*_*3*_ = 19.22) and gender ratios (female: *C*_*1*_ = 0.56, *C*_*2*_ = 0.70, *C*_*3*_ = 0.53). Similar to study 1, we examined whether the reported social distance differed across the three conditions. We found that participants reported the largest social distance (*M* = 7, *SD* = 0) with the stranger bystander (i.e., long social distance condition). As in the study 1, we then performed the Bonferroni-corrected t-test to examine the differences between each of the two conditions. The results showed the social distance between the key bystander present in the materials and the participants was significantly different across the three conditions (close friend vs. stranger: *t* (53) = 31.41, *p* < .001, Cohen’s *d* = 6.07; causal friend vs. stranger, *t* (45) = 11.48, *p* < .001, Cohen’s *d* = 2.37; close friend vs. causal friend, *t* (98) = 12.88, *p* < .001, Cohen’s *d* = 2.60). These results indicated the manipulation of social distance was effective (see Table 1).

#### Effects of social distance on embarrassment

As described in the study1, we conducted a one-way ANOVA of the embarrassment scores with social distance as a between-subjects factor in the study 2. Analyses revealed that the main effect of social distance was significant, *F* (2, 148) = 38.95, *p* < .001, *η*_*p*_^2^ = 0.34. Further analysis showed significant differences between each pairing condition, *ps* < 0.05 (Bonferroni corrected). Consistent with the results of the study 1, participants reported the highest level of embarrassment when facing a stranger (*M* = 4.36, *SD* = 1.05), the second-highest level when facing a casual friend (*M* = 3.87, *SD* = 0.81), and the lowest level when facing a close friend (*M* = 2.73, *SD* = 1.03).

#### The mediation model considering both the fear of negative evaluation and state attachment security

**Fig. 2:**
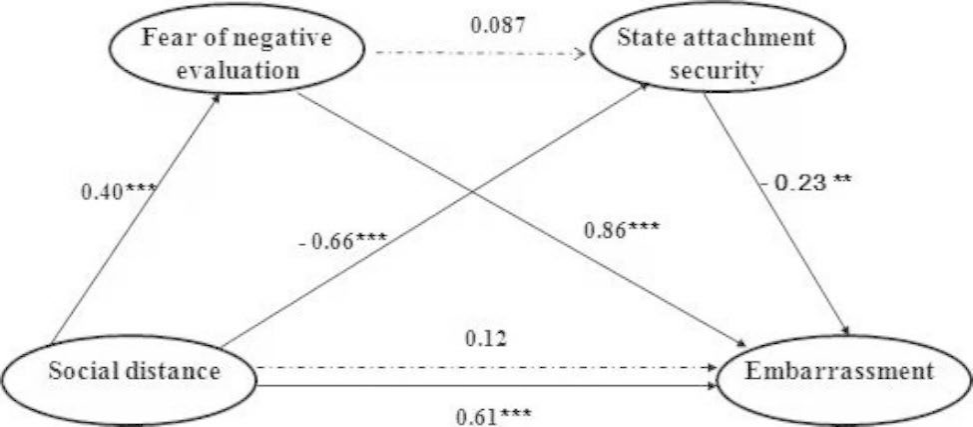
**The first mediation model.** The model showed that the fear of negative evaluation and state attachment security were two parallel mediators behind the effect of the social distance between bystander and protagonist on embarrassment. The numbers above the paths represent normalized coefficients. *** *p* < .001. ** *p* < .01

Here, we investigated whether the fear of negative evaluation and state attachment security function as mediators in the effect of social distance on embarrassment. To address this, a full mediation model was used, with the social distance reported by participants as the independent variable, the fear of negative evaluation as the first mediator, the state attachment security as the second mediator, and the embarrassment of participants as the dependent variable. Note that we used measurement of social distance rather than its categorical value in the model, because social distance is supposed to be a continuous variable that depends on different bystanders as well as on the participants’ own personal characteristics. All data were standardized. We used the PROCESS macro introduced by Hayes [29] and set the bootstrapping samples to 10,000. The mediation model analysis revealed that state attachment security and fear of negative evaluation functioned as two parallel mediators. Specifically, the fear of negative evaluation mediated the effect of social distance on embarrassment (*β* = 0.34, *SE* = 0.069, 95% CI = [0.22, 0.48]); sate attachment security mediated the effect of social distance on embarrassment (*β* = 0.15, *SE* = 0.053, 95% CI = [0.06, 0.27]). However, the serial mediating pathway was not significant. Besides, the effect of social distance on embarrassment became not significant after controlling for the mediating effects of the fear of negative evaluation and state attachment security (see Fig. 2).

**Fig. 3:**
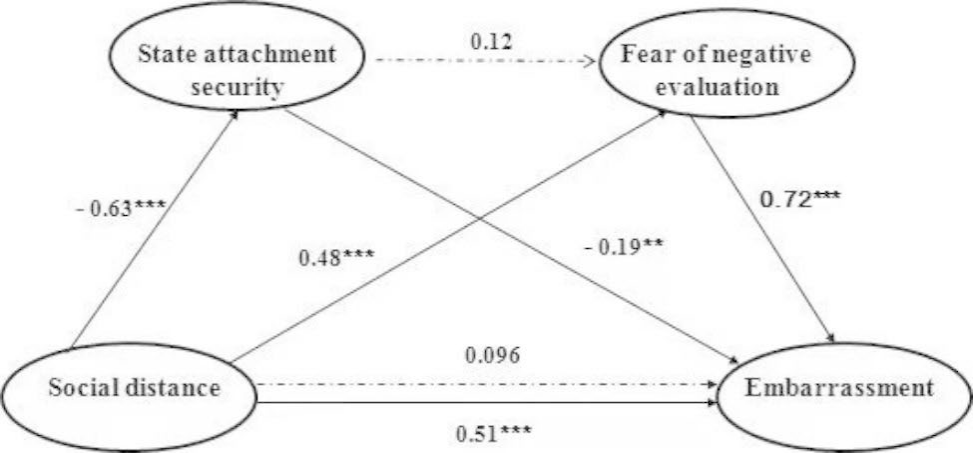
The second mediation model. The model further verified that the fear of negative evaluation and state attachment security mediated the influence of the social distance between bystander and protagonist on embarrassment in parallel. The numbers above the paths represent normalized coefficients. *** p < .001. ** p < .01

To further examine the potential interplay between state attachment security and fear of negative evaluations, a second full mediation model was developed. In this model, the state attachment security was the first mediator, and the fear of negative evaluation was the second mediator. This model also found state attachment security and fear of negative evaluation worked as two parallel mediators. Specifically, the state attachment security mediated the effect of social distance on embarrassment (*β* = 0.12, *SE* = 0.045, 95% CI = [0.05, 0.22]), as well as fear of negative evaluation (*β* = 0.35, *SE* = 0.081, 95% CI = [0.18, 0.50]), while the serial mediation pathway was not significant too (see Fig. 3). The correlation matrix of measured variables in the study 2 can be found in Table 2.

**Table 2:** Correlation matrix of measured variables of the study 2

	SD	FNE	SAS	Embarrassment
SD	1			
FNE	0.400**	1		
SAS	− 0.625**	− 0.177*	1	
Embarrassment	0.507**	0.795**	− 0.382**	1

Note: SD = Social distance; FNE = Fear of negative evaluation;

SAS = State attachment security. **p < .01, * *p* < .05

Note that, fitting indexes of the two parallel mediating models were present in supplement material for those interested, and the two models consistently showed that the effect of social distance on embarrassment could be well addressed by the parallel mediation model considering both state attachment security and the fear of negative evaluation.

## Discussion

The current study investigated whether and how the social distance between the protagonist and bystanders affected embarrassment. Study 1 found that participants felt the highest level of embarrassment when facing strangers, the second-highest level when facing casual friends, and the lowest level when facing close friends. These findings were consistent with those of a study showing that embarrassment was affected by the presence of different bystanders, with participants in the stranger-bystander condition reporting more experiences of embarrassment than those in the friend-bystander condition [10], and further demonstrated that the level of participants’ embarrassment varied with changes in the social distance (short social distance vs. medium social distance vs. long social distance). Briefly, study 1 confirmed that individuals’ embarrassment was influenced by the social distance between them and bystanders and that greater social distance resulted in greater embarrassment feelings.

Study 2 further investigated how the social distance affected embarrassment. The mediation analysis showed that both the fear of negative evaluation and state attachment security were mediators in this effect. According to social evaluation theory [14], in an embarrassing situation, people may worry about negative evaluations by bystanders, and such worry causes them more embarrassed. Considering that people close to the participants are more likely to have a robust positive impression of them, we expected that the embarrassed participants would worry less about the negative evaluation from these close people. The result supported the hypothesis above, and provided the evidence for Miller’s theory that fear of negative evaluation is a major cognitive process behind embarrassment. On the other hand, considering that people’s feelings of embarrassment are related to their real-time insecurity state [15, 16] and the presence of close others could temporarily increase the security of the flustered individual [19], it can be convinced that an increase in security feelings brought by close others may reduce individuals’ embarrassed feelings. Our research supported the view above and further revealed a close relationship between embarrassment and state attachment security, which is reasonable but easily overlooked.

Moreover, the two full mediation models consistently showed state attachment security and fear of negative evaluation mediated the effects of the social distance on embarrassment in parallel. The results suggested that the occurrence of an embarrassing event simultaneously activates protagonists’ attachment system and their fear of negative evaluation. That is, the cognitive process of seeking attachment for security may be an independent and fundamental cognitive process behind embarrassment. We think this cognitive process of seeking attachment for security may be important for our understanding of the distinctive features between different negative self-conscious emotions. Specifically, in a state of shame, the presence of close others made people avoid being touched [30]; whereas in a state of embarrassment, the presence of close others made people seek security from them. That is, feeling shame or embarrassment may depend on whether the attachment security system is activated by the awkward events, and by inducing the cognitive process associated with attachment seeking, a state of shame could be transferred to feelings of embarrassment, a temporary and less damaging emotion.

Notably, the influences of different bystanders during an embarrassing event are complex and whether the current findings are moderated by varying social contexts needs to be further explored. In the present study, we matched the age and gender of the bystander (e.g., male or female) present to each participant to control for the relevant confounding effects. Nevertheless, it needs to be investigated how our observations hold across different genders and ages by manipulating these bystander factors. Second, embarrassment is culturally sensitive and there may be cultural differences in bystander effects on embarrassment, which should also be explored in future research. Moreover, the current study focused on embarrassment and did not measure other emotional states (e.g., shame, sadness, or anger). Future research could measure other emotions, such as shame, along with embarrassment to better understand the impact of bystanders on the embarrassment.

Briefly, the current findings revealed that the social distance between bystanders and protagonists systematically influenced the embarrassment of protagonists and this effect occurred via two parallel pathways, i.e., by increasing the fear of negative evaluation and by reducing state attachment security. The findings not only indicate the unique role of bystander characteristics on embarrassment, but also suggest that fearing negative evaluation and seeking attachment for security are potential cognitive process behind the unique self-conscious emotion, embarrassment.

## Electronic supplementary material

Below is the link to the electronic supplementary material.

Supplementary Material 1

## Data Availability

The datasets generated or analysed during the current study are available in the repository, https://osf.io/8gwrb/?view_only=503116b865464ba7ba0222d77cfcce2b. Experimental materials in this study are included in this published article and its supplementary information files.
