# Supplementary material for: Social distance of bystanders affects people’s embarrassment via changing fear of negative evaluation and feelings of attachment security
Source: BMC Psychol. 2023 May 18;11:161. doi: 10.1186/s40359-023-01201-7 (PMC10193680; doi:10.1186/s40359-023-01201-7)
Supplement: Supplementary file 1 — Supplementary Material 1 [file 40359_2023_1201_MOESM1_ESM.doc]

**Supplementary materials**

**S1 Ten embarrassing scenarios used in this study**

1. You go to the bathroom in a hurry, and then come out with a close friend/casual friend/stranger reminding you that you just entered the wrong bathroom.

2. On the way to the classroom, a close friend/casual friend/stranger reminds you that you forgot to zip your pants.

3. While crossing the street, you accidentally bump into a close friend/casual friend/stranger walking across the street. He/she drops something in his/her hand, and you apologize to him/her.

4. In a restaurant, you are seen by a close friend/casual friend/stranger as you are cutting a steak, accidentally tipping the plate and the steak falls on your lap.

5. When you go to the buffet and take unconsciously too much food, you notice a close friend/casual friend/stranger and others staring at you.

6. When you are looking for a table in the canteen and your soup spills out and stains the clothes of a close friend/casual friend/stranger.

7. On the way to the library, a close friend/casual friend/stranger sees you tearing your pants while squatting down to tie your shoelaces.

8. You're singing at the top of your voice while walking around campus, and then you realize that a close friend/casual friend/stranger is looking at you for your singing out of tune.

9. You were spotted by a close friend/casual friend/stranger throwing a soda bottle into the trash and accidentally throwing it outside.

10. You are talking to a close friend/casual friend/stranger and accidentally spit on their face.

**S2 Table 1 Fit indices of the two models (without serial mediating pathway)**

|  | Model 1 | Model 2 |
| --- | --- | --- |
| GFI | .995 | .995 |
| AIC | 19.61 | 19.58 |

*Note*:

GFI = goodness-of-fit index; AIC = Akaike information criterion.

**S3 Cross-condition comparison of gender rates**

We conducted ANOVAs on gender rates across the three conditions (short, medium and long conditions) in Study 1 and Study 2. The results showed no gender rates differences across the three conditions in Study 1, χ2 (2) = 3.16, p = 0.21 and Study 2, χ2 (2) = 0.99, p = 0.61.
